# Causal effects for neurodegenerative diseases on the risk of myocardial infarction: a two-sample Mendelian randomization study

**DOI:** 10.18632/aging.205909

**Published:** 2024-06-07

**Authors:** Jianing Chi, Jiaman Hu, Ningxia Wu, Hua Cai, Cailong Lin, Yingying Lai, Jianyu Huang, Weihua Li, Peng Su, Min Li, Lin Xu

**Affiliations:** 1 Department of Geriatric Cardiology, General Hospital of Southern Theater Command, Guangzhou, China; 2 Branch of National Clinical Research Center for Geriatric Diseases, Chinese PLA General Hospital, Guangzhou, China; 3 Guangzhou Key Laboratory of Cardiac Rehabilitation, Guangzhou, China; 4 The First School of Clinical Medicine, Southern Medical University, Guangzhou, China; 5 School of Public Health, Guangdong Pharmaceutical University, Guangzhou, China; 6 Graduate School, Guangzhou University of Chinese Medicine, Guangzhou, China

**Keywords:** neurodegenerative diseases, Alzheimer’s disease, dementia with Lewy bodies, Parkinson’s disease, multiple sclerosis

## Abstract

Several studies have demonstrated a correlation between neurodegenerative diseases (NDDs) and myocardial infarction (MI), yet the precise causal relationship between these remains elusive. This study aimed to investigate the potential causal associations of genetically predicted Alzheimer’s disease (AD), dementia with Lewy bodies (DLB), Parkinson’s disease (PD), and multiple sclerosis (MS) with MI using two-sample Mendelian randomization (TSMR). Various methods, including inverse variance weighted (IVW), weighted median (WM), MR-Egger regression, weighted mode, and simple mode, were employed to estimate the effects of genetically predicted NDDs on MI. To validate the analysis, we assessed pleiotropic effects, heterogeneity, and conducted leave-one-out sensitivity analysis. We identified that genetic predisposition to NDDs was suggestively associated with higher odds of MI (OR_IVW=1.07, OR_MR–Egger=1.08, OR_WM=1.07, OR_weighted mode=1.07, OR_simple mode=1.10, all P<0.05). Furthermore, we observed significant associations of genetically predicted DLB with MI (OR_IVW=1.07, OR_MR–Egger=1.11, OR_WM=1.09, OR_weighted mode=1.09, all P<0.05). However, there was no significant causal evidence of genetically predicted PD and MS in MI. Across all MR analyses, no horizontal pleiotropy or statistical heterogeneity was observed (all P>0.05). Additionally, results from MRPRESSO and leave-one-out sensitivity analysis confirmed the robustness of the causal effect estimations for genetically predicted AD, DLB, PD, and MS on MI. This study provides further support for the causal effects of AD on MI and, for the first time, establishes robust causal evidence for the detrimental effect of DLB on the risk of MI. Our findings emphasize the importance of monitoring the cardiovascular function of the elderly experiencing neurodegenerative changes.

## INTRODUCTION

As the #1 cause of mortality and disability globally, cardiovascular diseases (CVD) especially myocardial infarction (MI), claim an estimated 17.9 million lives yearly, which poses an increasing burden on health and society [1]. The impact of CVD is predominantly observed in the elderly, as evidenced by the landmark Framingham Heart Study, revealing an eight-fold increase in MI incidence among older individuals compared to their younger counterparts [2]. Despite advancements in interventional therapies that have enhanced the quality of life for elderly MI patients, it still constitutes 30% of global mortality [3]. Hence, it is crucial to delve into the risk factors and pathogenesis of MI to enhance preventive measures and control.

As the world’s population ages, the incidence of neurodegenerative diseases (NDDs) is projected to surge by over 50% by 2030 [4, 5], resulting in a disease burden of 167.9 million disability-adjusted life years, second only to CVD [6]. Predominant age-related NDDs encompass Alzheimer’s disease (AD), dementia with Lewy bodies (DLB), Parkinson’s disease (PD), and multiple sclerosis (MS). The neuronal deterioration and degeneration associated with these diseases lead to motor dysfunction and cognitive decline, imposing a substantial burden on individuals, families, and society [7]. In recent years, there has been a growing interest in researching the association between NDDs and CVD [8–10].

The brain and the heart, integral components of the organism, share a profound functional connection, constantly interacting and mutually influencing each other at the pathophysiological level. The intricate brain-cardiac axis arising from this connection plays a pivotal role in the development of numerous neurological and cardiovascular diseases. However, the shared risk factors between the two complicate and cast controversy on studying their causality. Compelling evidence already exists that cardiovascular diseases contribute to the progression of neurodegenerative diseases. In a study involving over 4000 Chinese patients with PD, coronary heart disease emerged as one of the most prevalent comorbidities contributing to patient mortality [11]. Chinese population-based cohort studies suggested an association between coronary artery disease and PD [12]. Moreover, according to Newman AB and Roberts RO, there is a clear correlation between coronary heart disease and a heightened risk of AD [13, 14]. The Rotterdam Study indicated that unrecognized MI was linked to the risk of AD, whereas recognized MI was not [15]. Simultaneously, a higher Framingham cardiovascular risk score was associated with an increased risk of relapses, disability, and disease-modifying therapy escalation in patients with MS over a 5-year follow-up [16]. Several investigations have also explored the reciprocal effects of neurodegenerative alterations on cardiovascular disease [17]. Nevertheless, these studies were observational, prone to various measurement errors, potential biases, and confounders, making it challenging to distinguish the true causes and consequences between NDDs and MI.

The Two Sample Mendelian Randomization (TSMR), an emerging analytical approach, employs genetic variants as proxies for exposures to assess the causal impact of those exposures on outcomes [18–20]. Grounded in Mendel’s law of segregation, which posits that allele pairs segregate during gamete formation and randomly unite at fertilization [21], TSMR utilizes single nucleotide polymorphisms (SNPs) within genes as instrumental variables (IVs). This strategic use of IVs helps bypass traditional confounding factors and mitigates the influence of reverse causality between exposures and outcomes [22–25].

Consequently, to investigate the potential causality of genetic predisposition to NDDs concerning the risk of myocardial infarction, we conducted a TSMR analysis.

## MATERIALS AND METHODS

### Study design

**Figure 1:**
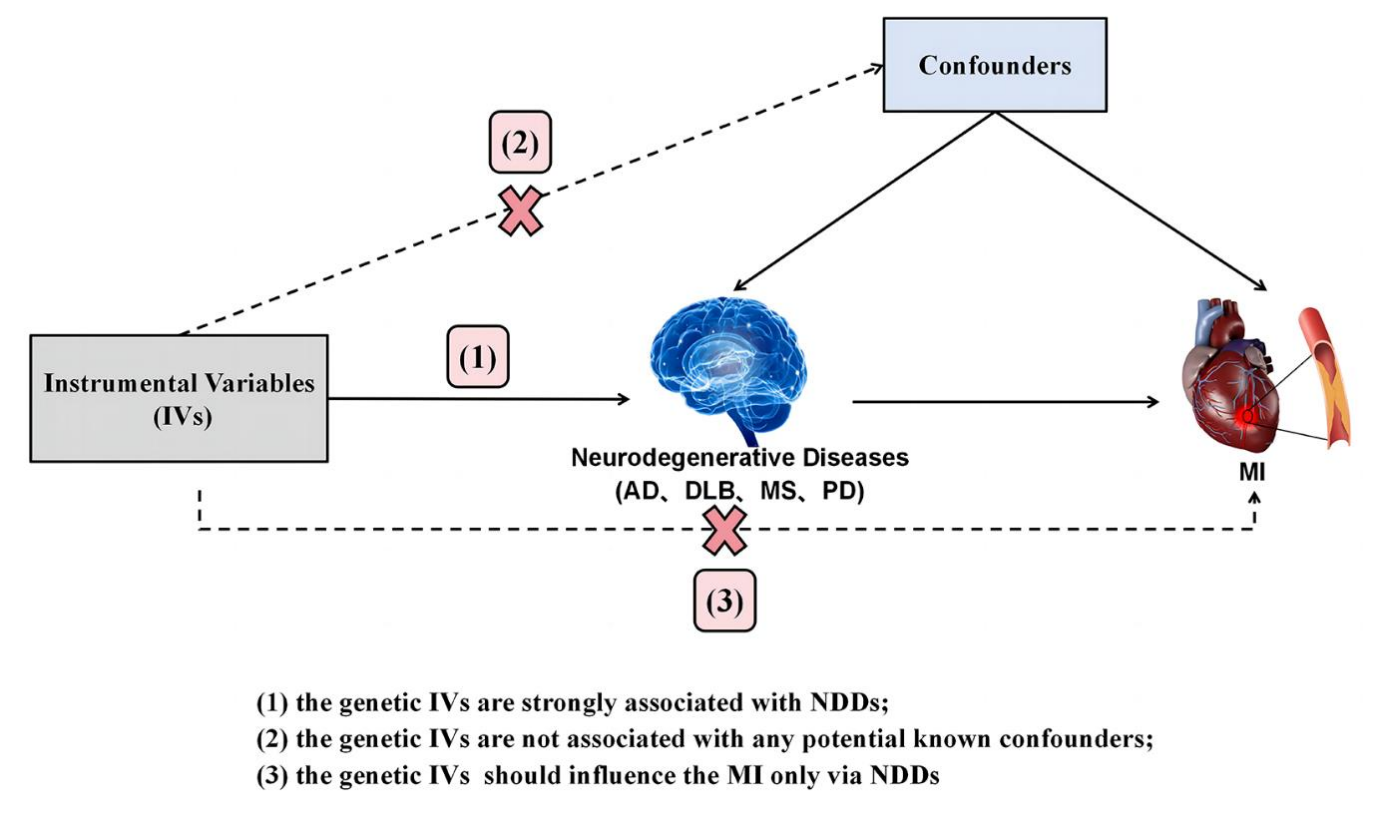
**Diagram of the TSMR study for the association between NDDs (AD, DLB, PD, MS) and the risk of MI.** (1), (2) and (3) are the research hypotheses. TSMR, two sample Mendelian randomization; NDDs, neurodegenerative diseases; AD, Alzheimer’s disease; PD, Parkinson’s disease; DLB, dementia with Lewy bodies; MS, multiple sclerosis; MI, myocardial infarction.

The current investigation employed a Two-Sample Mendelian Randomization (TSMR) study design guided by three hypotheses: (1) a strong association exists between genetic instrumental variables (IVs) and neurodegenerative diseases (NDDs); (2) the genetic IVs do not exhibit associations with any known potential confounders; (3) the genetic IVs exert influence on Myocardial Infarction (MI) solely through their impact on NDDs. The conceptual schematic of this MR study is depicted in Figure 1. Specifically, genetic variants robustly linked to neurodegenerative diseases, including Alzheimer’s Disease (AD), Dementia with Lewy Bodies (DLB), Parkinson’s Disease (PD), and Multiple Sclerosis (MS), were utilized as IVs, with MI considered as the outcome. Therefore, TSMR served as the primary statistical approach to explore causal associations between each IV-exposure (AD, DLB, PD, MS) and IV-outcome pairing.

### Data sources

Summary-level GWAS data for the associations of NDDs-associated SNPs with MI were derived from the Coronary Artery Disease Genome-Wide Replication and Meta-analysis plus The Coronary Artery Disease (CARDIoGRAMplusC4D) consortium, which involved 43,676 MI cases and 128,199 controls [26]. Meanwhile, we analyzed four common neurodegenerative disorders as exposures. Genetic variants associated with AD were obtained from a FinnGen biobank analysis in a total European population of 2,670 AD patients and 214,871 cognitively normal controls. The genome-wide association data for DLB were obtained from publicly available GWAS summary statistics, including 2,591 DLB patients and 4,027 control individuals [27]. The most recent and largest GWAS of PD involving 482,730 European participants, conducted by the International Parkinson’s Disease Genomics Consortium (IPDGC), was used for the summary statistics for PD outcome. The study detected and reported more than 15 million SNPs in 33,674 PD patients and 449,056 controls [28]. Genetic instrumental variables for MS were extracted from the International Multiple Sclerosis Genetics Consortium (IMSGC), covered 14,498 MS cases and 24,091 control, all of whom were of European ancestry [29]. All datasets included in the current study are summarized in Table 1.

**Table 1:** Detailed information of the studies and datasets used for Mendelian randomization analyses.

**Phenotypes**	**Sample size**	**SNP**	**Consortium of study**
Myocardial infarction	171,875	9,289,492	CARDIoGRAMplusC4D
Alzheimer’s disease	27,541	16,380,466	FinnGen
Dementia with Lewy bodies	6,618	7,593,175	Chia R, ect.
Parkinson’s disease	482,730	17,891,936	IPDGC
Multiple sclerosis	38,589	156,632	IMSGC

CARDIoGRAMplusC4D, Coronary Artery Disease Genome-Wide Replication and Meta-analysis plus The Coronary Artery Disease; IPDGC, International Parkinson’s Disease Genomics Consortium; IMSGC, International Multiple Sclerosis Genetics Consortium; SNP, single nucleotide polymorphism.

### Selection of instrumental variables (IVs)

To ensure the quality of genetic instruments, we implemented a rigorous series of quality control techniques. Firstly, we selected Single Nucleotide Polymorphisms (SNPs) strongly associated with AD, DLB, PD, and MS (P < 5×10^-8) at the genome-wide significance level. Secondly, to ensure the independence of each SNP and exclude variants in strong Linkage Disequilibrium (LD), we performed a clumping procedure with standard parameters (R^2 < 0.001, window size = 10,000 kb). Thirdly, SNPs associated with MI (P < 5×10^-8) were excluded using the GWAS Catalog. SNPs with a minor allele frequency < 0.01 were also eliminated. To harmonize exposure and outcome datasets, palindromic SNPs and ambiguous SNPs with non-concordant alleles were excluded [30]. Before MR analysis, F statistics of AD, DLB, PD, and MS were calculated to assess the strength of the IVs, with a threshold of F statistics > 10 indicating relatively strong estimated effects in the MR analyses [31, 32].

### Statistical analysis

We employed multiple methods, including Inverse Variance Weighted (IVW), MR-Egger regression, Weighted Median (WM), Weighted Mode, and Simple Mode, to estimate the impact of neurodegenerative diseases (NDDs) on myocardial infarction (MI) susceptibility. The IVW method, a primary and widely utilized approach in Mendelian Randomization (MR) analysis, summarized the weighted average of Wald ratio estimates of causal effects for each genetic variant. This method showcased the impact of each Single Nucleotide Polymorphism (SNP) at a standardized log-transformed exposure level, providing robust causal estimates [21]. In instances of weak instrumental variables (IVs), the MR–Egger method was employed to obtain less biased causal estimations. We further corroborated the results using the WM, which calculates the causal estimate by utilizing the reciprocal of its variance and generally exhibits greater power with a positive causal effect [33]. The Weighted Mode assigned causal estimates for each genetic variation based on the reciprocal of its variance, while the Simple Mode estimated the causal effect considering each genetic variant individually. Subsequently, causal effect estimates were calculated and converted to odds ratios (ORs).

Following the MR analysis, a series of sensitivity analyses, including assessments of heterogeneity, pleiotropy, and leave-one-out analysis, were conducted to evaluate the validity and robustness of the outcomes. Cochran’s Q statistic assessed the heterogeneity of the IVs, MR-Egger regression evaluated the directional pleiotropy of instrumental variables by comparing the deviation of the MR-Egger intercept from zero [34]. Additionally, the MR-PRESSO global test was performed to evaluate the existence of horizontal pleiotropy, and outlier IVs were identified through the MR-PRESSO outlier test [35]. Finally, a leave-one-out analysis was executed for each SNP to identify potential MI-associated SNPs that might influence the causal effect and to detect outliers [35].

All statistical analyses were performed using the “TwoSampleMR” and “MR-PRESSO” package in R software (version 4.2.2). A P-value less than 0.05 was considered statistically significant.

### Data availability statement

Publicly available datasets were analyzed in this study. This data can be found here: website of the ieu open gwas project (https://gwas.mrcieu.ac.uk/). The datasets generated during and/or analysed during the current study are available from the corresponding author on reasonable request.

## RESULTS

### IVs selection

Through the aforementioned series of selecting for SNPs associated with exposure and removing LD processes, 6, 6, 23 and 49 SNPs associated with AD, DLB, PD and MS were obtained, respectively. We further identified 6 SNPs shared by AD and MI, there were no SNPs associated with AS, the 6 SNPs were used as IVs, and there were no palindromic SNPs. There were also no SNPs associated with MI in 6 SNPs shared by DLB and MI, the 6 SNPs were used as IVs, and there were no palindromic SNPs. Meanwhile, we identified 23 SNPs shared by PD and MI, there were one SNPs associated with MI (rs144814361), the 22 SNPs were used as IVs, and there were six palindromic SNPs (rs10451230, rs12934900, rs329647, rs35265698, rs4613239, rs823106). In the 49 SNPs shared by MS and MI, there were four SNPs associated with AS (rs12210359, rs9277535, rs9277535, rs2857700, rs9263823), the 45 SNPs were used as IVs, and there was six palindromic SNP (rs1131265, rs12296430, rs1359062, rs212405, rs9736016, rs9989735). The F-statistics were all >10 in the analyses of this study, indicating no evidence of weak IV bias was found. Thus, these IVs are proven appropriate estimates of the normal impact of exposure and outcome. Detailed information about all the IVs is provided in Supplementary Table 1.

### AD do have causal effects on MI

**Figure 2:**
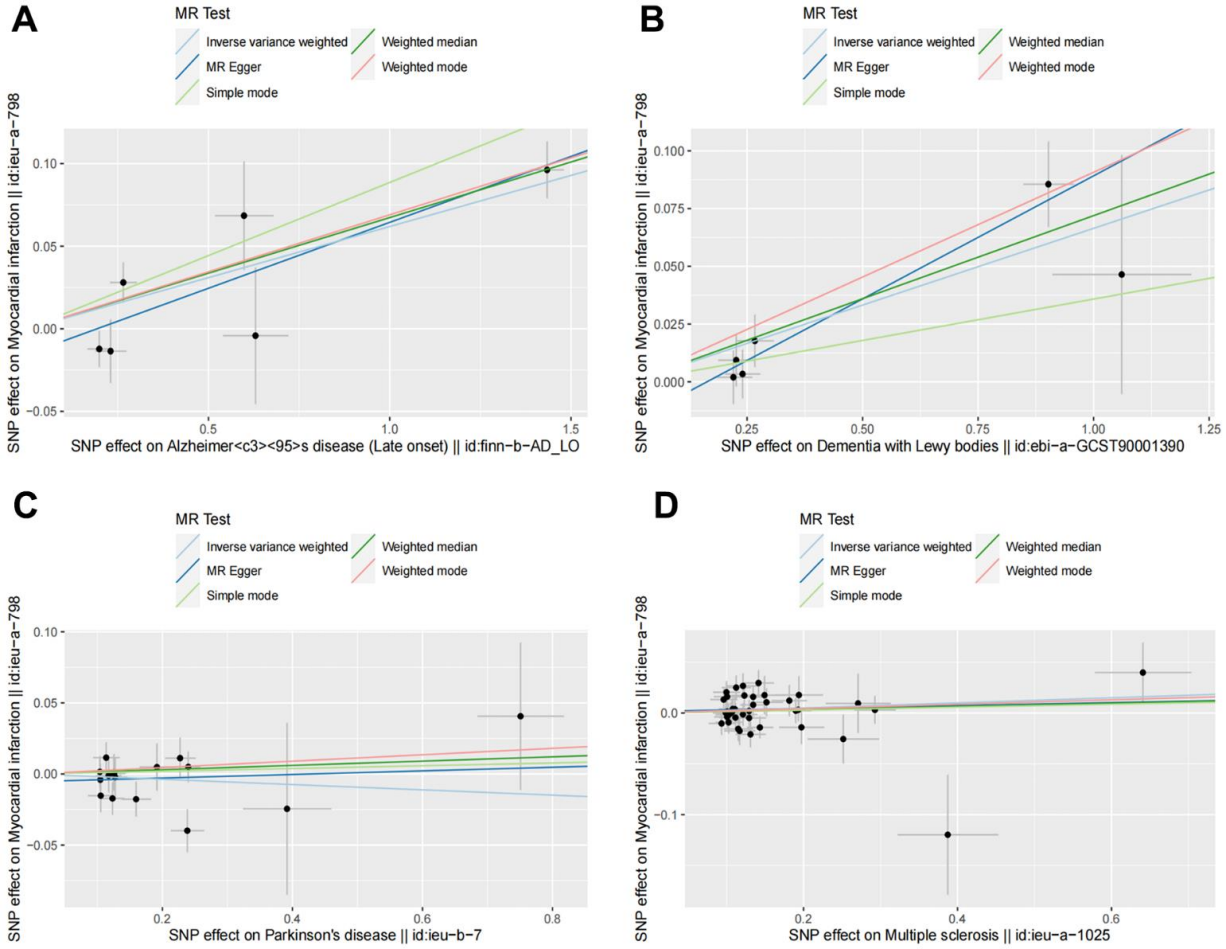
**Scatter plots for MR analyses of the causal effect of NDDs phenotypes on MI.** The vertical axis is the effect of SNP on MI and the horizontal axis is the effect of SNP on NDDs phenotypes. (**A**) AD-MI. (**B**) DLB-MI. (**C**) PD-MI. (**D**) MS-MI. Analyses were conducted using the conventional IVW, WM, MR-Egger, simple mode and weighted mode methods. The slope of each line corresponds to the estimated MR effect per method. MR, Mendelian randomization; IVW, inverse variance weighted; WM, weighted median.

**Figure 3:**
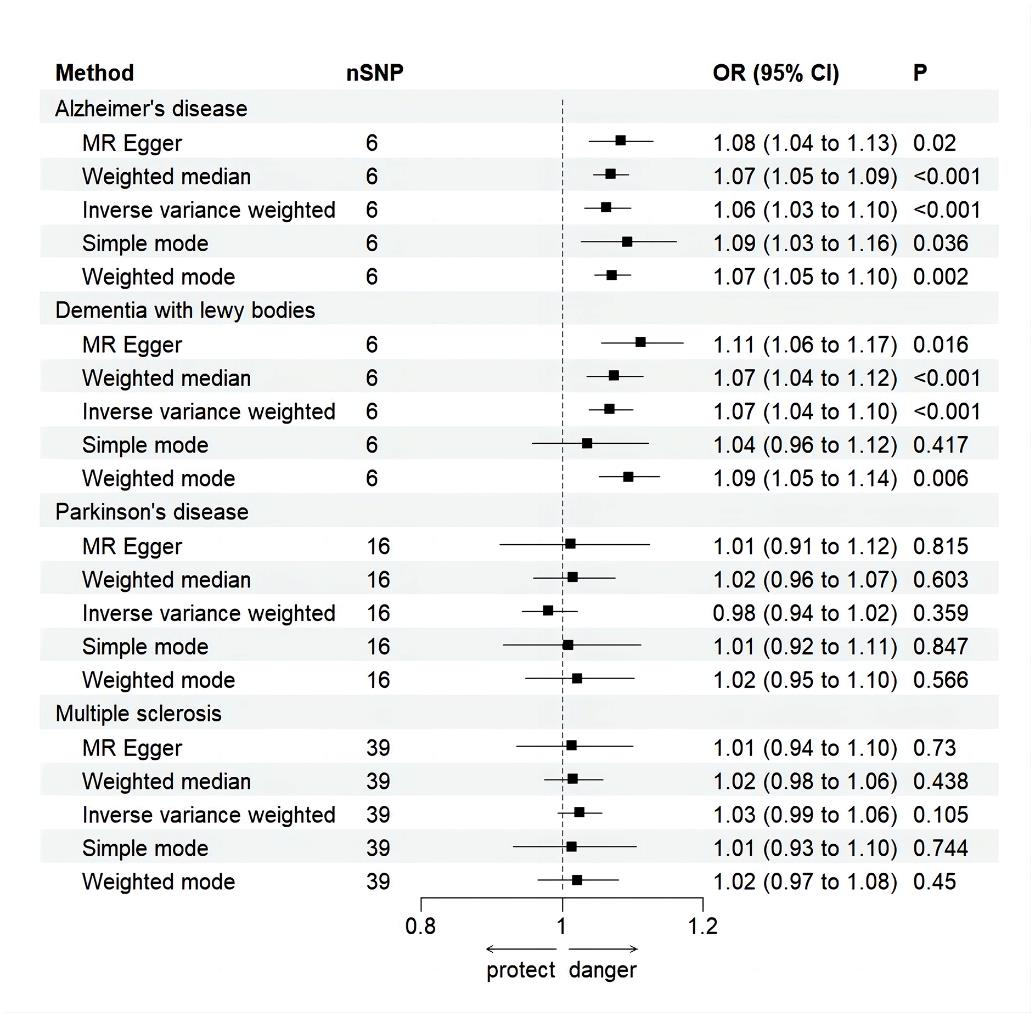
**Forest plot for MR analysis causal effect of NDDs phenotypes on MI.** Five methods: random-effects IVW, MR–Egger, WM, simple mode and weighted mode. IVW was used as the main method to analyze the causal relationship. OR are scaled to per genetically predicted 1 log-odds unit increase in the liability to NDDs. The standard line is the “X = 1” dotted line. P-value less than 0.05 is considered statistically significant. SNP, single nucleotide polymorphism; OR, odds ratio; CI, confidence interval; Error bars represent the 95% CI for the estimates.

**Figure 4:**
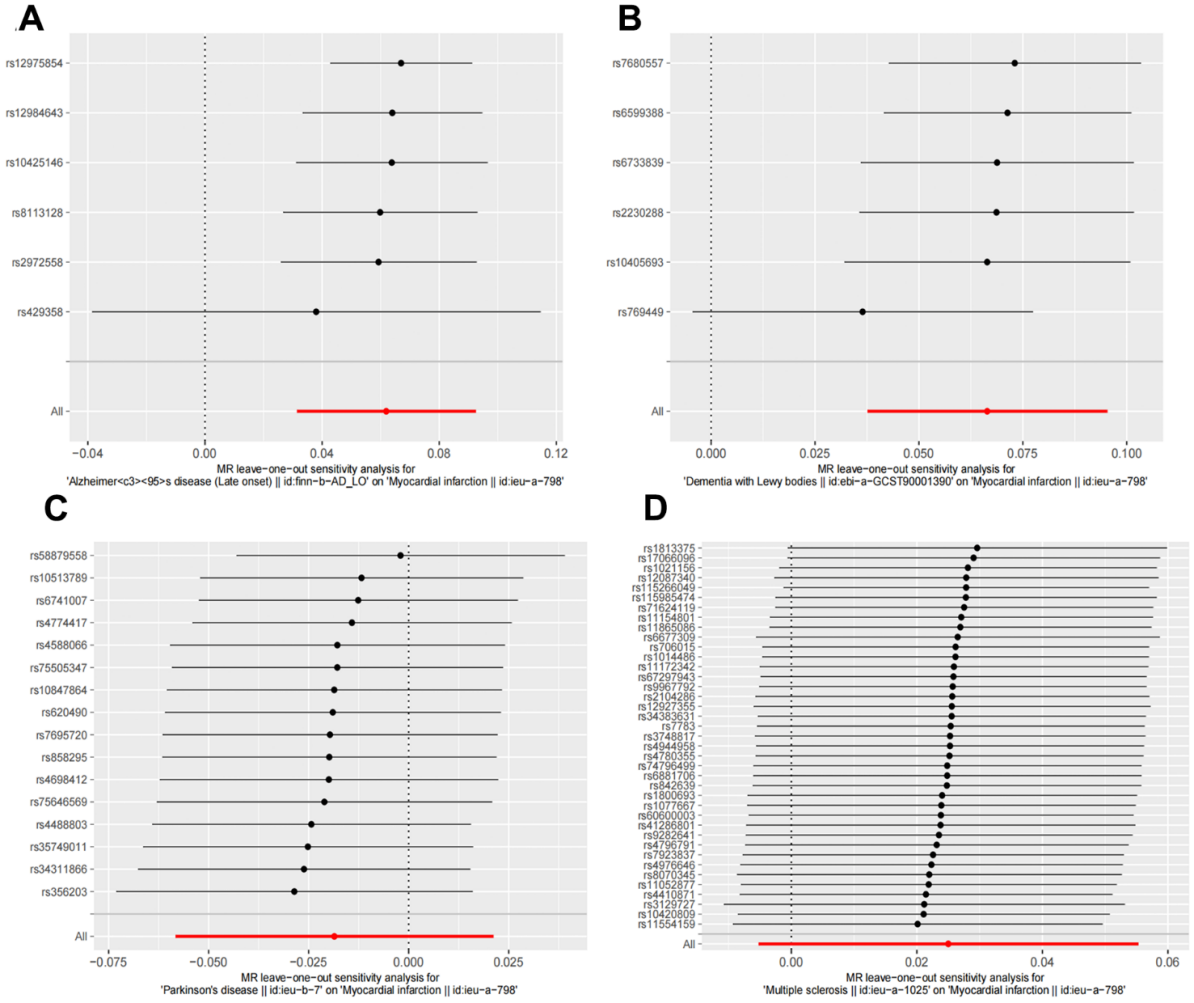
**Leave one out sensitivity analysis between NDDs phenotypes and MI.** The results revealed no apparent outliers among all the SNPs. (**A**) AD-MI. (**B**) DLB-MI. (**C**) PD-MI. (**D**) MS-MI. NDDs, neurodegenerative diseases; AD, Alzheimer’s disease; MS, multiple sclerosis; PD, Parkinson’s disease; DLB, dementia with Lewy bodies; IVW, Inverse variance weighted; PD, Parkinson’s disease; AD, Alzheimer’s disease.

Random effects IVW results showed that genetically predicted AD occurrence was significantly associated with a higher incidence of MI (OR: 1.07, 95% CI: 1.03-1.10, P < 0.001). Likewise, results from all of the MR–Egger (OR: 1.08, 95% CI: 1.04-1.13, P = 0.039), WM (OR: 1.07, 95% CI: 1.05-1.10, P < 0.001), weighted model (OR: 1.07, 95% CI: 1.05-1.10, P = 0.004) and simple mode (OR: 1.10, 95% CI: 1.04-1.17, P = 0.028) demonstrated a significant relationship between the two (Figures 2A, 3). Cochran’s Q statistic (MR-IVW) and Rucker’s Q statistic (MR–Egger) showed no heterogeneity in the MR analysis of AD versus MI (PIVW =0.052, PMR-Egger =0.077). Similarly, the MR-Egger intercept (P = 0.367) and MR-PRESSO global test (P = 0.363) showed a low likelihood of horizontal pleiotropy (Table 2). The leave-one-out analysis and MR PRESSO outlier test revealed no apparent outliers among the SNPs (Table 2 and Figure 4A).

**Table 2:** Sensitivity test of the Mendelian randomization analysis between NDDs phenotypes and MI.

**NDDs** **phenotypes**	**Methods**	**Heterogeneity test**		**Horizontal pleiotropy**		**MR-PRESSO**	
		**Cochran’s Q**	**p**	**Egger_intercept**	**p**	**p of global test**	**p of Outlier test**
AD				-0.015	0.299	0.363	NA
	IVW	7.762	0.101				
	MR-Egger	10.520	0.062				
DLB				-0.017	0.143	0.376	NA
	IVW	1.801	0.772				
	MR-Egger	5.107	0.403				
PD				-0.005	0.534	0.502	NA
	IVW	15.195	0.365				
	MR-Egger	15.636	0.407				
MS				0.002	0.786	0.089	NA
	IVW	46.362	0.139				
	MR-Egger	46.456	0.163				

p-value less than 0.05 is considered statistically significant. NDDs, neurodegenerative diseases; AD, Alzheimer’s disease; DLB, dementia with Lewy bodies; PD, Parkinson’s disease; MS, multiple sclerosis; IVW, Inverse variance weighted.

### Causal effects of DLB on MI

It’s worth noting that we found evidence of a positive association between DLB and MI based on the IVW method (OR: 1.07, 95% CI: 1.04-1.10, P < 0.001), indicating that a genetically determined increased DLB engagement could increase the risk of MI. Besides, the results of the MR-Egger (OR: 1.11, 95% CI: 1.06-1.17, P = 0.016), WM (OR: 1.07, 95% CI: 1.04-1.12, P < 0.001) and weighted model (OR: 1.09, 95% CI: 1.05-1.14, P = 0.005) all demonstrated a significant relationship between the two. In addition, the simple mode results (OR: 1.04, 95% CI: 0.96-1.12, P = 0.416) shared similar patterns of effect, although the statistical significance was absent (Figures 2B, 3). The P-values for the IVW-Q test and the MR–Egger-Q test were 0.402 and 0.772, respectively, suggesting a lack of horizontal pleiotropy among the SNPs, thus providing robust results. Meanwhile, no directional pleiotropy was suggested by the MR–Egger intercept with an intercept value of -0.017 and p-value of 0.143. The leave-one-out and MR-PRESSO analysis (global test: P = 0.376) also indicated that there was no horizontal pleiotropy, so there were no outliers for any IVs (Table 2 and Figure 4B).

### No causal relationship is observed between MS and PD and MI

For the MS, there was no evidence of heterogeneity as measured by the Q statistics (Q_IVW = 46.36, P = 0.139; Q_MR–Egger = 46.45, P = 0.163). MR–Egger intercept analyses and MR-PRESSO global test found no evidence for any pleiotropy in the MS-MI associations (P_MR-Egger intercept = 0.786, P_MR-PRESSO = 0.089), indicating the stability of our analysis (Table 2). Additionally, through the leave-one-out sensitivity analysis, we observed minimal alterations in the overall results upon systematically excluding each SNP, which reinforces the robustness and reliability of our findings (Figure 4C). Multiple MR methods, including the IVW method (OR: 1.03, 95% CI: 0.99-1.06, P = 0.105), showed that the trend of the effect of MS on MI risk was consistent that the risk of MI tended to increase when the genetically determined PD engagement increased despite the association was nonsignificant (Figures 2C, 3). Similar findings were also observed in the causal effect estimation between PD and MI. There was no evidence of heterogeneity (P_IVW = 0.364, P_MR–Egger = 0.407) and any pleiotropy (P_MR-Egger intercept = 0.534, P_MR-PRESSO = 0.502) in the PD-MI relationship (Table 2). Besides, there were no outliers for any IVs by the leave-one-out analysis (Figure 4D). The results of the IVW, MR–Egger, median-based and maximum likelihood methods demonstrated in Figures 2D, 3 were implied that there was no significant causal association, and the effect pattern varied for the different methods (OR_IVW: 0.98, 95% CI: 0.94– 1.02, P = 0.357; OR_MR Egger: 1.01, 95% CI: 0.91–1.12, P = 0.815; OR_WM: 1.02, 95% CI: 0.96–1.07, P = 0.603; OR_weighted mode: 1.02, 95% CI: 0.95–1.10, P = 0.566; OR_simple median: 1.02, 95% CI: 0.91–1.11; P = 0.847).

## DISCUSSION

In the current study, we utilized large GWAS datasets to investigate the causality between NDDs (AD, DLB, PD, MS) and MI. We discovered suggestive genetic evidence supporting the causal associations of genetic liability to AD and DLB with an elevated risk of MI. Although the relative risk increase may not appear substantial, it still holds significant epidemiological and clinical implications. However, our results indicated that PD and MS were not genetically correlated with MI. It cannot be ruled out, though, that they may be related at other levels beyond genetics.

For Alzheimer’s Disease (AD) and Myocardial Infarction (MI), we have identified a causal relationship between them, aligning with the findings of previous studies [36]. AD, the most prevalent irreversible neurodegenerative diseases (NDDs), is characterized by neuronal degeneration, loss of neurons, brain atrophy, senile plaques (SP), and neurofibrillary tangles (NFT) [37]. The pathogenesis of AD is multifactorial and involves complex interactions among various environmental and genetic factors. The currently recognized theories include the genetic, beta-amyloid (Aβ), tau, and neuroinflammatory theories [38]. As a primary pathological hallmark of AD, the deposition of Aβ and tau can promote vascular dysfunction through oxidative stress-mediated eNOS interactions and agonist-mediated disruption of Akt activation, which are involved in atherosclerosis formation and the development of MI [39]. Additionally, Aβ acts as an inflammatory stimulus, activating complement, inducing changes in glial cell responses, and releasing inflammatory proteins such as cytokines, chemokines, and adhesion molecules, all of which play a role in the development of MI [40]. Furthermore, NFT, caused by the aggregation of abnormally phosphorylated tau proteins, represents another significant injury in AD. This interference with cardiac neuromodulation and vascular function contributes to coronary artery spasm or constriction, thereby increasing the risk of MI [41, 42]. Moreover, a previous meta-analysis of cytokines in AD revealed significantly increased levels of inflammatory factors such as TNF-α, IL-1β, NF-κB, and TLR2 in AD patients [43, 44]. Numerous studies have suggested that inflammatory responses constitute a primary pathogenic factor in MI [45–47]. Additionally, AD and MI share common risk factors, including hypercholesterolemia, hypertension, diabetes mellitus, obesity, smoking, and physical inactivity [48]. These factors induce oxidative stress, vascular endothelial dysfunction, and inflammatory responses, collectively leading to MI [49]. Most importantly, genetically, the apolipoprotein E (apoE) 4 gene is involved in the development of cognitive impairment by regulating interferon-related gene expression in the choroid plexus [50]. Meanwhile, it has been reported to have a significant effect on cholesterol metabolism and lipid transport, thus becoming one of the risk factors for MI [51]. In addition, TREM2, which is associated with AD, Clusterin (CLU, also known as apolipoprotein J, ApoJ), which is associated with the complement signaling pathway, and complement receptor 1 (CR1) genes are also involved in the modulation of the inflammatory response [52], further increasing the risk of MI. However, the specific mechanisms underlying the causal relationship between AD and the risk of MI are still in the preliminary stages and require further exploration through larger clinical studies and basic experiments.

What is noteworthy is that our study, for the first time, provides robust causal evidence of the detrimental effect of DLB on the risk of MI. DLB is a type of neurodegenerative diseases (NDDs) with a prevalence second only to Alzheimer’s Disease (AD), stemming from the formation of Lewy bodies and manifesting primarily in a wide range of cognitive, neuropsychiatric, sleep, motor, and autonomic symptoms, often with a poor prognosis [53, 54]. To the best of our knowledge, the mechanism between DLB and MI has not been studied. One possibility is that the accumulation of Lewy bodies leads to mitochondrial damage and fragmentation, ultimately inciting the cascade of cellular apoptosis and death, which may cause MI [55, 56]. Additionally, the largest risk factor for developing DLB is age, with most cases becoming clinically evident at approximately 70 years of age. The risk of developing DLB is higher in men than in women, which is consistent with the trend observed in MI. Another possible mechanism is that DLB exhibits the same accelerated deposition of extracellular Aβ peptide aggregated plaques as AD [57], causing microangiopathy such as vessel wall rupture and cortical microinfarcts, which in turn increases the risk of MI. Moreover, genetic alterations are observed in the microtubule-associated protein tau, scavenger receptor class B (SR-BI), and apoE genes of DLB [58, 59]. Studies show that abnormally phosphorylated and aggregated tau proteins are associated with cardiomyocyte dysfunction and cell death, leading to a high risk of MI. Genetic variations in SR-BI and apoE, which are lipoprotein receptors, significantly impact lipid levels, thereby contributing to the development of coronary artery disease and even MI. Both Zhang et al. and Mark Fuller et al. conducted animal experiments demonstrating that the deletion of SR-BI in apoE knockout or hypomorphic mice resulted in occlusive coronary atherosclerosis, spontaneous myocardial infarction, cardiac dysfunction, and premature death [60, 61]. Besides, genetic studies have revealed that some patients with DLB have mutations in the α-synuclein gene (SNCA), which acts as a lipid-binding protein that interacts with phospholipids and fatty acids to participate in the process of lipophagy, impairing a variety of subcellular functions and leading to dysregulation of lipid metabolism, which in turn may contribute to the development of MI [62]. Most importantly, there is growing evidence of an inflammatory response in the blood of DLB patients, such as elevated expression of chronic inflammatory factors such as Tumor Necrosis Factor-α (TNF-α), Interleukin-1β (IL-1β), and Interleukin-6 (IL-6) compared to controls [63]. While MI is usually caused by coronary artery obstruction, chronic inflammation is associated with the development of atherosclerosis and plaque destabilization [64], which may be one of the mechanisms by which genetically predicted DLB increases the risk of MI. The correlation between DLB and MI risk is intriguing, and further investigation is required to pinpoint these plausible confounding factors and clarify the complex association among them.

For MS and PD, the results reported by several previous observational epidemiological studies on the associations between them and MI were inconsistent and mixed. A Canadian study tracked 44,452 MS patients and 220,849 age-, sex-, and geographically matched controls for a decade, which reported a reduced incidence of ischemic heart disease in the MS population, particularly among those aged 60 years and older [65]. Another community-based study found lower ischemic heart disease-related mortality in PD patients [66]. However, a recent study using data from the National Health Insurance database in Taiwan suggested that newly diagnosed PD patients may face a statistically significant increased risk of subsequent MI [17]. Additionally, a multi-database study indicated an elevated risk of MI among female MS patients [67]. The hypothesized possibility that immobilization and physical inactivity in MS patients increases the risk of MI [68]. Besides, immunomodulatory treatments such as interferon β have been found to be associated with the risk of CVD in MS patients [69]. In genetic terms, the causality found may be due to alterations in Human Leukocyte Antigen (HLA), CD40, and PINK1/Parkin genes in patients with MS and PD, which modulate the body’s immune-inflammatory response and mitochondrial autophagy, thereby influencing the occurrence of myocardial infarction [70–73]. It’s essential to acknowledge potential biases in these observational studies, such as confounding variables and subjective viewpoints. To more accurately establish causal associations between PD, MS, and MI, our study employed the TSMR approach, enhancing internal validity by using robust instrumental variables. Despite the strength of our TSMR design and the precision achieved through diverse analytical techniques, the study has limitations. The non-causal relationship we derived between MS, PD and MI may be due to the relatively small sample size, which affected the stability of the results. It is therefore necessary to conduct further studies using larger datasets to improve the validity of the study.

Our study’s primary strength lies in the TSMR design, which utilizes robust IVs to mitigate confounding and bias associated with reverse causality in observational research. Simultaneously, the precision and repeatability of the findings are enhanced by the variety of analytical techniques employed in this investigation. Additionally, the analysis demonstrates the robustness of our results, with no significant evidence of heterogeneity and pleiotropy. However, our study has several limitations. Firstly, the dataset exclusively represents European populations, potentially limiting the generalizability of our findings to other ancestral groups. Also, a stratified discussion of the important factor of gender is not possible due to the lack of gender-related variables in the dataset. Thirdly, due to the small size of the screened sample in this study, it may lead to lower statistical validity and the risk of false-negative results. Besides, our results show that multiple SNPs in each phenotype of NDDs are associated with myocardial infarction. Nevertheless, the proteins involved in these SNPs were not further explored in this study and need to be investigated in future. Moreover, for the causal relationship between NDDs and MI, genetic variation can only explain part of it, and more fundamental experiments are needed to verify and explore the specific related mechanisms.

## CONCLUSIONS

Our study provides further support for the causal effects of AD on MI and, for the first time, establishes robust causal evidence for the detrimental effect of DLB on the risk of MI. Our findings emphasize the importance of monitoring the cardiovascular function of the elderly experiencing neurodegenerative changes.

## Supplementary Material

Supplementary Table 1
