# Supplementary material for: Causal effects for neurodegenerative diseases on the risk of myocardial infarction: a two-sample Mendelian randomization study
Source: Aging (Albany NY). 2024 Jun 7;16(11):9944–58. doi: 10.18632/aging.205909 (PMC11210233; doi:10.18632/aging.205909)
Supplement: Supplementary Table 1 [file aging-16-205909-s001.docx]

**Supplementary Table 1: Summary characteristics of selected genetic variants associated with neurodegenerative diseases phenotypes used as instrumental variables.**

| NDDs phenotypes | SNP | position | EA | OA | EAF | β | se | *P-value* | F | sample size |
| --- | --- | --- | --- | --- | --- | --- | --- | --- | --- | --- |
| AD | rs10425146 | 44730553 | T | G | 0.0179 | 0.6298 | 0.0892 | 1.70E-12 | 49.84757835 | 27541 |
| AD | rs8113128 | 45700657 | C | A | 0.0477 | 0.5981 | 0.08 | 7.92E-14 | 55.89025507 | 27541 |
| AD | rs429358 | 45411941 | C | T | 0.1551 | 1.434 | 0.0449 | 1.00E-200 | 1019.938725 | 27541 |
| AD | rs2972558 | 45356141 | T | C | 0.7068 | 0.2652 | 0.0357 | 1.09E-13 | 55.17966609 | 27541 |
| AD | rs12975854 | 45120592 | T | C | 0.337 | 0.199 | 0.0321 | 5.65E-10 | 38.42948362 | 27541 |
| AD | rs12984643 | 14353096 | G | T | 0.1571 | 0.2301 | 0.0419 | 4.06E-08 | 30.15599428 | 27541 |
| DLB | rs10405693 | 45326664 | T | C | 0.6551 | 0.266854 | 0.0399007 | 2.26E-11 | 44.71519594 | 171875 |
| DLB | rs2230288 | 155206167 | T | C | 0.9791 | 1.06082 | 0.149491 | 1.28E-12 | 50.34102311 | 171875 |
| DLB | rs6599388 | 939087 | T | C | 0.6998 | 0.220396 | 0.0399821 | 3.54E-08 | 30.37700467 | 171875 |
| DLB | rs6733839 | 127892810 | T | C | 0.6203 | 0.22653 | 0.0385412 | 4.16E-09 | 34.53581575 | 171875 |
| DLB | rs7680557 | 90763360 | A | C | 0.5119 | 0.240262 | 0.0371284 | 9.73E-11 | 41.86262343 | 171875 |
| DLB | rs769449 | 45410002 | A | G | 0.8767 | 0.902123 | 0.0538205 | 4.65E-63 | 280.8696341 | 171875 |
| PD | rs10451230 | 16035225 | T | A | 0.5666 | -0.096 | 0.0175 | 4.42E-08 | 30.09293655 | 482730 |
| PD | rs10513789 | 182760073 | G | T | 0.1978 | -0.1596 | 0.0219 | 3.18E-13 | 53.10993196 | 482730 |
| PD | rs10847864 | 123326598 | T | G | 0.339 | 0.1274 | 0.0179 | 9.81E-13 | 50.65601184 | 482730 |
| PD | rs12934900 | 30923602 | T | A | 0.6352 | 0.1215 | 0.0184 | 4.33E-11 | 43.60287346 | 482730 |
| PD | rs329647 | 133764666 | C | G | 0.7038 | -0.1133 | 0.0178 | 1.94E-10 | 40.51520267 | 482730 |
| PD | rs34311866 | 951947 | C | T | 0.1869 | 0.2272 | 0.0231 | 7.97E-23 | 96.73661688 | 482730 |
| PD | rs35265698 | 32561334 | G | C | 0.159 | -0.2 | 0.0303 | 3.93E-11 | 43.56853199 | 480593 |
| PD | rs356203 | 90666041 | T | C | 0.6133 | -0.2398 | 0.0178 | 3.01E-41 | 181.4916101 | 482730 |
| PD | rs35749011 | 155135036 | A | G | 0.0219 | 0.7508 | 0.0659 | 5.02E-30 | 129.8003607 | 482730 |
| PD | rs4488803 | 58218352 | A | G | 0.3728 | -0.1136 | 0.0199 | 1.08E-08 | 32.5873249 | 482730 |
| PD | rs4588066 | 40672964 | A | G | 0.339 | 0.1046 | 0.0178 | 4.45E-09 | 34.53198671 | 482730 |
| PD | rs4613239 | 169119609 | G | C | 0.1213 | 0.1784 | 0.0248 | 6.21E-13 | 51.746924 | 482730 |
| PD | rs4698412 | 15737348 | A | G | 0.5596 | 0.1258 | 0.0168 | 7.05E-14 | 56.07133798 | 482730 |
| PD | rs4774417 | 61993702 | A | G | 0.7137 | 0.1052 | 0.0192 | 4.63E-08 | 30.02114298 | 482730 |
| PD | rs58879558 | 44159631 | C | T | 0.2505 | -0.2383 | 0.025 | 1.36E-21 | 90.85864756 | 482730 |
| PD | rs620490 | 16697579 | G | T | 0.2992 | -0.1174 | 0.019 | 6.46E-10 | 38.1792324 | 482730 |
| PD | rs6741007 | 135537119 | G | T | 0.497 | -0.1233 | 0.0175 | 2.09E-12 | 49.64188412 | 482730 |
| PD | rs75505347 | 40885549 | T | C | 0.0209 | 0.3917 | 0.0674 | 6.12E-09 | 33.77423732 | 482730 |
| PD | rs75646569 | 60345424 | G | T | 0.1252 | 0.1916 | 0.0266 | 5.62E-13 | 51.88310236 | 482730 |
| PD | rs7695720 | 77183300 | C | A | 0.2356 | -0.1255 | 0.0208 | 1.53E-09 | 36.40482791 | 482730 |
| PD | rs823106 | 205656453 | C | G | 0.8549 | -0.1492 | 0.0239 | 4.10E-10 | 38.97086496 | 482730 |
| PD | rs858295 | 23245569 | G | A | 0.3708 | -0.1039 | 0.0176 | 3.83E-09 | 34.85009451 | 482730 |
| MS | rs1014486 | 159691112 | C | T | 0.4335 | 0.10075 | 0.0165979 | 1.16E-09 | 36.84353674 | 38589 |
| MS | rs1021156 | 79575804 | C | T | 0.7575 | -0.115113 | 0.0186355 | 5.60E-10 | 38.15435676 | 38589 |
| MS | rs10420809 | 10727014 | T | C | 0.7288 | 0.121332 | 0.0210163 | 7.96E-09 | 33.32849364 | 38589 |
| MS | rs1077667 | 6668972 | T | C | 0.2142 | -0.151862 | 0.0208049 | 3.54E-13 | 53.27757581 | 38589 |
| MS | rs11052877 | 9905690 | G | A | 0.3642 | 0.0998453 | 0.0170901 | 5.37E-09 | 34.13056953 | 38589 |
| MS | rs11154801 | 135739355 | A | C | 0.366 | 0.102557 | 0.0170438 | 2.35E-09 | 36.20555829 | 38589 |
| MS | rs11172342 | 58187758 | T | G | 0.3298 | -0.121332 | 0.0178497 | 9.46E-12 | 46.20259418 | 38589 |
| MS | rs1131265 | 119222456 | C | G | 0.8037 | 0.173953 | 0.0220662 | 1.97E-15 | 62.14214449 | 38589 |
| MS | rs115266049 | 30431011 | A | G | 0.0121 | 0.387301 | 0.0649399 | 2.37E-09 | 35.56734899 | 38589 |
| MS | rs11554159 | 18285944 | A | G | 0.2697 | -0.1415 | 0.0192663 | 2.58E-13 | 53.9378627 | 38589 |
| MS | rs115985474 | 30055179 | T | C | 0.0337 | -0.251537 | 0.0459673 | 4.34E-08 | 29.94216879 | 38589 |
| MS | rs11865086 | 30130493 | A | C | 0.4376 | -0.0934903 | 0.0164939 | 1.77E-08 | 32.12653469 | 38589 |
| MS | rs12087340 | 85746993 | T | C | 0.0867 | 0.19721 | 0.0286886 | 5.13E-12 | 47.25161387 | 38589 |
| MS | rs12296430 | 6503500 | C | G | 0.189 | 0.128393 | 0.0204191 | 3.62E-10 | 39.5354778 | 38589 |
| MS | rs12927355 | 11194771 | T | C | 0.3221 | -0.193097 | 0.018079 | 8.19E-27 | 114.0721781 | 38589 |
| MS | rs1359062 | 192541472 | G | C | 0.1836 | -0.162119 | 0.0221213 | 1.84E-13 | 53.70615143 | 38589 |
| MS | rs17066096 | 137452908 | G | A | 0.2294 | 0.131028 | 0.0190212 | 5.91E-12 | 47.44930884 | 38589 |
| MS | rs1800693 | 6440009 | C | T | 0.3979 | 0.134531 | 0.016723 | 6.92E-16 | 64.71334547 | 38589 |
| MS | rs1813375 | 28078571 | T | G | 0.4695 | 0.143234 | 0.0165781 | 5.75E-18 | 74.64491389 | 38589 |
| MS | rs2104286 | 6099045 | C | T | 0.2785 | -0.189794 | 0.0194055 | 7.61E-23 | 95.65171783 | 38589 |
| MS | rs212405 | 159470559 | T | A | 0.6228 | 0.139762 | 0.017738 | 1.43E-15 | 62.07924167 | 38589 |
| MS | rs3129727 | 32681820 | T | C | 0.0232 | -0.640801 | 0.0622141 | 6.85E-25 | 106.083101 | 38589 |
| MS | rs34383631 | 60793330 | T | C | 0.3961 | 0.105261 | 0.0167604 | 5.69E-10 | 39.44061838 | 38589 |
| MS | rs3748817 | 2525665 | C | T | 0.3588 | -0.129272 | 0.0181572 | 1.33E-12 | 50.68607618 | 38589 |
| MS | rs41286801 | 92975464 | T | C | 0.1441 | 0.181488 | 0.0225487 | 7.92E-16 | 64.77841285 | 38589 |
| MS | rs4410871 | 128815029 | C | T | 0.7175 | 0.112435 | 0.0187022 | 1.98E-09 | 36.14058226 | 38589 |
| MS | rs4780355 | 11347858 | C | T | 0.2895 | -0.10075 | 0.0182211 | 3.47E-08 | 30.57161749 | 38589 |
| MS | rs4796791 | 40530763 | C | T | 0.6354 | -0.0962189 | 0.0171367 | 1.81E-08 | 31.52418577 | 38589 |
| MS | rs4944958 | 71168073 | G | A | 0.7845 | -0.107059 | 0.0185652 | 8.69E-09 | 33.25252214 | 38589 |
| MS | rs4976646 | 176788570 | C | T | 0.3399 | 0.123102 | 0.0171486 | 1.04E-12 | 51.52884022 | 38589 |
| MS | rs60600003 | 37382465 | G | T | 0.1033 | 0.149282 | 0.0268082 | 2.53E-08 | 31.00680877 | 38589 |
| MS | rs6677309 | 117080166 | C | A | 0.1214 | -0.29267 | 0.0264562 | 1.45E-28 | 122.3711315 | 38589 |
| MS | rs67297943 | 138244816 | C | T | 0.2159 | -0.111541 | 0.0205287 | 4.83E-08 | 29.5204968 | 38589 |
| MS | rs6881706 | 35879156 | T | G | 0.2754 | -0.110647 | 0.0189559 | 4.87E-09 | 34.06967254 | 38589 |
| MS | rs706015 | 27014988 | G | T | 0.1819 | 0.129272 | 0.0212989 | 1.29E-09 | 36.8359672 | 38589 |
| MS | rs71624119 | 55440730 | A | G | 0.2448 | -0.116894 | 0.019729 | 2.70E-09 | 35.10361034 | 38589 |
| MS | rs74796499 | 88432328 | A | C | 0.0459 | -0.27079 | 0.041786 | 8.47E-11 | 41.99340934 | 38589 |
| MS | rs7783 | 16629189 | G | A | 0.3457 | 0.0989399 | 0.0171056 | 9.56E-09 | 33.45367246 | 38589 |
| MS | rs7923837 | 94481917 | A | G | 0.3882 | -0.10075 | 0.0170747 | 4.58E-09 | 34.81460214 | 38589 |
| MS | rs8070345 | 57816757 | C | T | 0.5467 | -0.134531 | 0.016723 | 5.43E-16 | 64.71334547 | 38589 |
| MS | rs842639 | 61095245 | A | G | 0.653 | 0.107957 | 0.0180902 | 1.70E-09 | 35.61167236 | 38589 |
| MS | rs9282641 | 121796768 | A | G | 0.0812 | -0.193921 | 0.0304428 | 1.74E-10 | 40.57494929 | 38589 |
| MS | rs9736016 | 118724894 | A | T | 0.3725 | -0.0971267 | 0.0173606 | 2.20E-08 | 31.29862767 | 38589 |
| MS | rs9967792 | 191974435 | C | T | 0.619 | 0.102557 | 0.0170285 | 1.80E-09 | 36.27064846 | 38589 |
| MS | rs9989735 | 231115454 | C | G | 0.1822 | 0.156149 | 0.0209432 | 7.84E-14 | 55.58656342 | 38589 |

Abbreviations: NDDs, neurodegenerative diseases; SNP, single nucleotide polymorphism; EA, effect allele; OA, other allele; EAF, effect allele frequency; se, standard error; AD, Alzheimer’s disease; LBD, Lewy body dementia;PD, Parkinson’s disease; MS, multiple sclerosis; MI,myocardial infarction.
